# 

**Published:** 1897-08

**Authors:** 


### ATLANTA MEDICAL AND SURGICAL JOURNAL.

VOLUME XIV NEW SERIES. ATLANTA, GA., AUGUST, 1897. No. 6.

